# Asylum Expenditure

**Published:** 1900-06-16

**Authors:** 


### ASYLUM EXPENDITURE.

"An Asylum Expert" writes: Our contemporary, the *Architect*, says that the outlay on county asylums is alarming, as it shows that brain diseases are increasing. We are not sure that the deduction is absolutely correct; but we are in perfect accord as far as the outlay is concerned. In many instances the cost of new asylums, and of additions to old ones, is far beyond what good accommodation can be provided for, and anything further than that must be looked on as extravagance. The *Architect's* words were called forth by the fact that the visitors of the Oxford Asylum are about to spend £35,629 on adding to and altering the building. Two hundred beds are to be added, and alterations are to be made in the chapel, kitchen, surgery, wards, &c. Assuming that the additions will cost £100 a bed, there would be £15,629 for the alterations and the furnishing. As things have gone lately in asylums we are not disposed to look on the sum as excessive, but it ought not to be exceeded. A complete asylum for 300 beds has been built for a similar sum.

